# Impact of COVID-19 response on public health literacy and communication

**DOI:** 10.34172/hpp.2022.01

**Published:** 2022-05-29

**Authors:** Namrata Hange, Arjola Musta Agoli, Maria Kezia Lourdes Pormento, Amrit Sharma, Manoj Reddy Somagutta, Nidhin Paikkattil, Amol Jadhav, Deepak Bethineedi, Pravin Pisude

**Affiliations:** ^1^Division of Research, Eurasian Cancer Research Council (ECRC), Chembur, Mumbai, Maharashtra, India; ^2^Tirana University of Medicine, Dept of Obstetrics & Gynecology, Rruga e Dibrës, Tirana, Albania; ^3^Ateneo School of Medicine and Public Health, Don Eugenio Lopez Sr. Medical Complex, Ortigas Ave, Pasig, 1604 Metro Manila, Philippines; ^4^Department of Community Health, Christian Medical College, Christian Medical College, Vellore, Tamil Nadu, India; ^5^Avalon University School of Medicine, 212 Churchill Hubbard Road Youngstown, OH, USA; ^6^Indian institute of technology, Kharagpur, West Bengal, India; ^7^Medical Operations, JV Gokal Charity Trust, Kasturi Building, 2nd Floor, 171/172, J Tata Road, Mumbai, Maharashtra, India; ^8^Andhra Medical College, Road King George Hospital, opp. Collector Office, Maharani Peta, Visakhapatnam, Andhra Pradesh 530002, India; ^9^ESIC Medical College and Hospital and SS Hospital, Sanath Naga Sanath Nagar Hyderabad, Telangana, India

**Keywords:** Health Promotion, Health communications, COVID-19, Prevention and control, Health literacy, Communication, Pandemics

## Abstract

With unaddressed challenges of pandemic with re-emergence of coronavirus disease 2019 (COVID-19) waves, public health literacy and communication have proved to be a prerequisite for effective communication as part of the control strategy. Hence this article addressed the impact of COVID-19 response policies on public health literacy. Considering the rapid transmission of COVID-19, taking lives needs urgent attention from the population›s perspective to be more vigilant about health information and incorporate that into their daily routines. To be responsible and resilient globally, governments and states are formulating different health policies and related plans to prevent and control the spread of the pandemic. This article has recommended short-term measures, including smart focused IEC targeted on vaccination and motivational sessions for health care workers and front line workers. Targeted Long-term measures included healthcare system reforms inclusive of resources, workforce, capacity building with particular focus on lifestyle measures addressing non-communicable disease prevention.

## Introduction

The global coronavirus disease (COVID-19) pandemic originated in Wuhan, China, in December 2019 and has claimed more than 4.2 million deaths so far.^1,2^ This disease is believed to be transmitted through direct, indirect, close contact with infected people through infected secretions such as saliva and respiratory secretions or droplets, through coughing, and sneezing.^3,4^ Until vaccine discovery, the healthcare system was dependent upon targeting standard operating procedures and restrictions on health behaviour for prevention.^5^ Despite safe and effective vaccines being looked at as a game-changing tool in transmission, World Health Organisation (WHO) still advises to be precautious and continue with wearing masks, hand washing, ensuring good ventilation, physically distancing, and avoiding crowds.^5-7^ The amplitude of individuals to access, process, and understand essential health information needed for making appropriate health decisions and outcomes has been reflected through health literacy. This requires knowledge, competencies, and or actions to revolve around the functional -interactive-critical cycle.^8,9^ Precise decisions-making through solid commitment and adherence to health behaviour are integral parts of public health literacy. These are essential in disease prevention and health promotion to maintain or improve quality of life.^9-11^Augmented health literacy boosts capability and motivation, finds solutions to health problems at the individual and community level, uses and addresses health problems through life courses. This process of underpinning health literacy involves empowerment at all levels.^11,12^ The emergence of COVID-19 has demonstrated the need for improved health literacy across populations throughout the healthcare system to fight this pandemic.^13^

Health literacy and communication channels have been concurrently saturated by a deluge of misinformation, further highlighting the need for policymakers and healthcare providers to provide reliable, accessible, and understandable information.^13-15^ COVID-19 pandemic signified alarming demand for healthcare system reforms considering this flooding of misinformation. This underestimation of health literacy and an abrupt bombardment of health policies addressing pandemics have created confusion and panic.^15^ However, a continuous influx of related resources on social media has become a double-edged sword.^16^ Given the context of the current health dilemma, health literacy is being looked upon as the primary focus to control the spread of transmission and promote healthcare systems preparedness to deal with future pandemics.^17^ Abel and McQueen reported the significance of health literacy in recognizing risk factors and adopting preventive measures.^18^ With the growing complexity of healthcare systems inclusive of unaddressed challenges of the pandemic with the re-emergence of COVID-19 waves, health literacy and communication are becoming a more necessary asset. Hence this article addresses the impact of COVID-19 response policies on public health literacy.

## Global response of COVID situation for lockdown

The subtle characteristics of the COVID-19 pandemic necessitated leaders from each country to impose some rules to curb the massive spread of the virus.^19,20^ Considering ongoing rapid transmission and increase in mortality, most countries issued stay-at-home orders for populations awaiting vaccine discovery referred to as “lockdown” after WHO declared COVID-19 a pandemic.^19,20^ The modelling analysis study found that well-timed lockdowns can split the peak of hospitalizations into two smaller distant peaks extending overall pandemic duration. The timing of lockdowns achieves a tunnelling effect on incidence to bypass rise and prevent pandemic caseloads from exceeding hospital capacity.^21^ A statistical analysis of incident cases, deaths, and intensive care units in Italy and Spain only reported a decline after introducing strict lockdown action.^22^ Likewise, lockdown in China reduced contact and ultimately bring-down transmission through isolation and quarantine procedures.^23^ Wuhan lockdown prevented 0.5-3 million illnesses and 18 000–70 000 deaths at the expense of negative impacts to the economy and restrictions to personal freedoms.^24^

## Social distancing

The respiratory virus is usually transmitted via viral-laden muco-salivary respiratory droplets, expelled during exhalation, speech, and violent respiratory events such as coughing and sneezing.^25^ Traditionally, respiratory disease transmission occurs through the droplet route for large droplets and aerosol for tiny droplets. Aerosol transmission assumes inhalation of pathogen-bearing droplets smaller than 5-10 microns in diameter.^26-28^ The idea of social distancing measures is primarily based on this classification with different interventions recommended for large droplets with the suspected airborne transmission.^27,28^ The spread of COVID-19 may be limited by population-based public health management adopted through social distancing, appropriate hand hygiene, and following the essential practice of covering the nose and mouth and avoiding crowds, poorly ventilated places.^27-30^ Moreover, real-time and retrospective analyses of the growth rate of COVID-19 cases and deaths have suggested that the epidemic eventually slowed after the implementation of concrete social distancing measures in Wuhan, Hong Kong, Europe, and the United States.^31-34^ Some studies recommended social distancing of at least 2 meter (6 feet) during this COVID-19 outbreak, while others believed it might not be adequate.^35-41^

## School closures

The United Nations Educational, Scientific, and Cultural Organization (UNESCO) 2020 estimated that 107 countries had implemented national school closures related to COVID-19, affecting 862 million children and young people, roughly half of the global student population.^41,42^ The evidence of reduced influenza incidence rates during influenza outbreaks after the early introduction of restrictive measures has made the government decide to undertake school closures.^42^ Considering higher viral shedding for a more extended period and inability to take proper precautionary measures to follow up on hygiene, children were considered as a major link in infection transmission.^43^ Possibly school closure was effective for infection control for outbreaks with low transmissibility but is not still validated for COVID with different transmission dynamics.^44,45^ With the shutdown of schools, one of the adaptive measures gaining traction is “online teaching,” with a spike in the use of platforms such as Zoom, Microsoft Teams, Google Meet, Got To Webinar, etc. UNICEF has reported 83% of countries used online platforms for education delivery. Policy measures taken by the governments to ensure learning continuity through broadcast or digital media allowed for potentially reaching a maximum of 69% of schoolchildren globally. Remaining children deprived of this facility due to lack of necessary technological assets at home or in need of the adopted policies.^46^ Social isolation in children was associated with loneliness, negative consequences on mental health, and other health-related behaviors for children.^42,46,47^

## Digital health literacy

The pandemic situation created a complex information environment that required people to access, navigate, understand, use, and critically evaluate information and services to support healthy and protective behaviors in the time of the pandemic. So, health literacy has become a detrimental factor in daily decision-making adopting health behaviour.^48^ Digital health literacy is the ability to obtain, process, understand, and communicate health-related information required to make informed health decisions in digital contexts and environments.^49-51^ This necessity becomes a core competence for navigating web-based information and health service environments within the realm of the COVID-19 pandemic and associated infodemic, with an overabundance of health information.^49-51^Sufficient health literacy mediates the effects of the social determinants of healthcare disparities, strengthening health literacy and reducing disparities to promote equity in health.^51-53^

## Prioritization of emergency services

In the aftermath of mass-casualty events, the overwhelming disaster critical resources and the critical upsurge in demand made response allocation challenging.^53,54^ The reorganization of services is achieved through prioritizing care triage, public-private partnership, and reorientation of referral pathways.^54,55^ Priority resource lists were developed, executed, and given a free hand to suppliers and pharmacies to create dynamic inventory assessment and coordinated re-distribution.^56^ Globally, two-thirds of countries included non-communicable disease services in national COVID-19 preparedness and response plans, including cardiovascular disease, cancer, diabetes, and chronic respiratory disease, including dental services, rehabilitation, and tobacco cessation activities in response plans.^55-57^

## Successful global example of health literacy and communication in COVID-19 control

Singapore›s extraordinary constant efforts concentrated through prompt response, advanced preparation, effective quarantine strategy, and effective communication to citizens regarding the COVID situation and precautions- received special recognition from the WHO. Effective communication strategies to combat fake news, Singapore’s approach has utilized marketing strategy through various social media channels including telegram, Twitter, and traditional ministerial media communications to broaden access for information, convey clarifications, and debunk falsehoods in an efficient and timely manner. COVID-19 chronicles consisted of burlesque series for COVID-19 updates addressing recommendations for prevention and seeking medical attention was another milestone. Singapore conducted simulations elucidating consolidated efforts of quarantine, social distancing, school and work closure in reducing the speed of COVID-19. This Two-pronged strategy not only addressed the scientific explanation behind these measures but also appealed to the recipient›s moral values. Safe distancing ambassadors and enforcement officers on the ground have reminded the public of wearing face masks at all times and maintaining a safe distance of at least one meter between individuals.^57^

Sweden was one of the few countries that decided to keep preschools and schools open. Though social distancing was encouraged in Sweden, no mandate was enforced regarding wearing face masks in children, possibly because of low incidence of severe COVID-19 among children. Children’s access and understanding of information about COVID-19 have given new insights on these aspects of children’s health literacy.^58,59^ Canada has effectively handled the challenge of reaching the countries diverse population. National press briefings dedicated to children and LEGO animations helped address children’s roles and convey the message. During the time of uncertainty, the opportunities created learning opportunities for children alongside parents and communities globally. Crucially, effective communications strategies helped jurisdictions in New Zealand by spearheading informal messages through official ministerial channels. Interestingly, the Prime minister with a degree in communications started the “Conversations through COVID” Zoom talk with the active involvement of children’s musicians, female indigenous scholars, and experts in mental health. Along with Facebook Live video streaming, other tools at disposal for communicating clearly and swiftly to citizens about what to expect and how to act were proved to be sources of reliable information.^59,60^

Israel, being a fully vaccinated country against COVID-19, has neared the threshold for herd immunity. High and low-tech vaccination scheduling approaches were used according to the target population›s needs and specifications with effective use of digital data analytics and roll out of fast vaccination, simplified age and professed based strategies. A customer-centric, highly centralized digital healthcare system with digital connectivity was instrumental in addressing vaccine hesitancy in people with rumours circulating, often via social media, over the efficacy and safety of the vaccines. Culturally adapted outreach efforts by influential religious leaders, answering questions, addressing fears, and adapting messages to diverse groups were embraced to address vaccine hesitancy.^61,62^

Taiwan has one of the lowest mortality burdens amongst high-income countries without a lockdown. Rapid and systematic implementation of control measures, effective border management, contact tracing, systematic quarantine/isolation of potential and confirmed cases, cluster control, the active promotion of mass masking, and meaningful public health communication proved instrumental in limiting pandemic spread in Taiwan. Taiwan’s emergency central epidemics command control coordinated with the national communications commission to generate materials for broadcast media, YouTube, memes, and even cartoon stickers of the health minister and effective communication addressed through Facebook, LINE, and a telephone hotline.^63^ The rare success story of Bhutan with fastest adult population vaccination campaigns covering 90% of the population with a strategy of community mobilization, meticulous authorized planning with limited resources of 30 000 strong force of citizen volunteers, dressed in bright orange, labelled as “Desuups.” This force of health workers helped reinforce public health messages such as encouragement of wearing masks and assisted in testing, surveillance, and contact tracing among Bhutan’s people.^64^

## Impact on health service delivery

A decline in the use of health services was reported in Wuhan in 2019-20, as a result of travel restrictions and longer prescriptions for drugs for non-communicable diseases.^65^ The visits to ambulatory practices in the United States were reported to have declined nearly at the same time.^66^ As in-person visits dropped, telehealth visits increased rapidly before plateauing. The relative decline in visits remains largest among procedural and surgical specialties and paediatrics with cancellation or postponement of thousands of elective procedures and outpatient appointments.^66-69^According to the WHO, prevention and treatment services for non-communicable diseases have been severely disrupted globally, translating high levels of heterogeneity in cumulative excess COVID-19 death rates among countries.^69-71^

Studies have reported high levels of stress and burnout among healthcare workers caring for COVID-19 patients^72,73^ This was associated with fear of exposure or transmission, self-reported anxiety/depression, the stress of not feeling valued.^73^ Across studies, there is solid evidence that nurses show poorer mental health outcomes - depression, anxiety, and posttraumatic stress disorder compared with medical doctors during the COVID-19 pandemic.^74^ That has reflected in high vaccine acceptance among healthcare professionals on priority while women reported slight hesitation considering pregnancy outcomes.^75,76^ Vaccine hesitancy represents a major barrier to implementing vaccination programs.^77,78^ A study addressing vaccine hesitancy reported vaccine novelty, wanting others to receive it first, and insufficient decision-making time, and determinable factors for refusal. Physicians, environmental services workers, and healthcare managers were more likely to accept vaccination vs. nurses.^79^ Underreporting of Coronavirus cases and deaths were happening in countries with a lack of access to health services deprived of laboratory testing and weak reporting systems and developing countries of challenges of testing and tracking as well.^80-82^ despite, being high health literacy in Germany, the population reported difficulties dealing with COVID-19 information. One of the studies reported participants felt well informed, but half of them had difficulties judging whether they could trust media information on COVID-19, possibly inadequacy or problematic communication.^83^ While a study conducted in Australia, people with inadequate health literacy had a poorer understanding of COVID-19 symptoms, were less able to identify behaviors to prevent infection, and experienced more difficulty finding information and understanding government messaging about COVID-19 than people with adequate health literacy.^84^ Countering the COVID-19 misinfodemic is a critical task for individual and public health. Healthcare professionals at all levels must strengthen their commitment to health literacy.^85^

## Way forward for public health literacy

Health literacy entails the acquisition of medical knowledge and the ability to understand the command to adopt healthy behaviors. The government, health information providers, health professionals, the media, and the general public all have a role in improving health literacy.^86^

## Acquisition

During the pandemic, social media platforms were a significant source of information.^86,87^ However, the information provided may not always be accurate. The WHO promoted digital health literacy to give wider access to health information at a lower cost.^86,87^ Individuals with better health literacy are likely to perceive health information better.^86,87^ But health literacy is context-dependent, and people with good health literacy may still find difficulties in certain situations. For example, individuals may find it difficult to distinguish scientific information from misinformed articles simply because they are unaware of medical jargon. Several researchers and organizations have presented guidelines, resources, digital tools, and strategies to combat this issue.^86-88^ Even WHO has set up the MythBuster to provide a quick guide on reliable information.^88,90^ and acknowledged the complexity of acquiring reliable information online. Still, proposed strategies or tools are still in development and one tool alone may not be completely effective in addressing misinformation. However, we must continue empowering and supporting health institutions to monitor, evaluate and respond to misinformation concerning the utilization of this data in vaccine acceptance.^89,90^ A study by Montagni et al. observed the association of intention to get the COVID-19 vaccine with the detection of misinformation and health literacy scores. Participants were more hesitant to get vaccinated than those who had difficulty identifying misinformation or had lower health literacy scores.^91^

## Understanding

**Figure 1:**
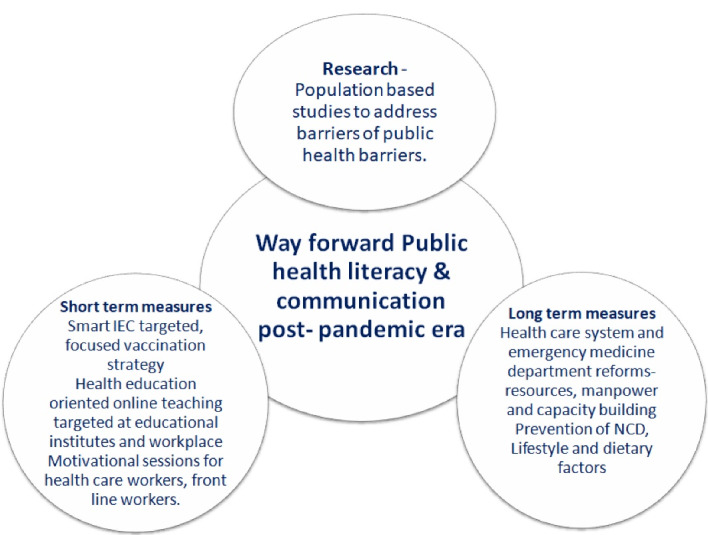
Recommendations to bridge barrier to achieve health literacy.

Health literacy does not depend solely on the acquisition of information. Health care personnel should be aware and receptive of the varied health care needs of the patients. This requires the health care provider to be proficient in several strategies and skills to deliver helpful information even to those with low levels of health literacy.^91-93^ One method is to develop the habit of the teach-back method. This entails the patient to reiterate the teachings of the health care provider as they understand them. This strategy can be employed in the clinical setting and during health education sessions, in schools, for employees, or in the community, both in-person and online. To further ensure comprehension, the information given should avoid medical jargon and be presented in small concrete steps.^94^The USA has developed a Health Literacy Universal Precautions Toolkit that recommends approaching each patient as if they had a low level of health literacy. However, this can be a challenging approach due to the limitations of the health provider.^92,93^ Health care providers and organizations should aim to know the health literacy level of the target population to deliver the best strategies to raise said levels. The interventions could include tailored awareness campaigns, community outreach, education, and training programs that address the community›s specific needs.^87-89^ Detailed recommendations bridge barrier to achieve health literacy has been described in Figure 1.

### Application

Outside of the clinical setting, health care providers or organizations find it challenging to promote positive health behaviors in individuals. Technological advancements may provide solutions in promoting health literacy. A study by Dunn and Hazzard identified several technologies addressing specific healthcare-related problems, such as wearable blood pressure monitoring devices, which improved blood pressure control and showed better medication adherence.^88,89^ Further research is needed to address public health barriers. The COVID-19 pandemic is still widespread and a significant threat to the lower health literacy population. The use of smart phones to reconnect with individuals in the outpatient setting may be influential, especially for those with chronic diseases or in rural areas, in developing healthcare solutions.^96^ Training primary care physicians and ancillary staff in these areas to incorporate mobile calls or short text messages may strengthen health care delivery.^87,94^

## Conclusion

COVID has undoubtedly altered the health care landscape and health information management. Timely updates comprehensible messages, including reliable information with meaningful joint effort and system support, increase public knowledge and confidence to deal with pandemics. This clear and credible communication advocated for people to be socially responsible and resilient. Marketing strategies through various social media channels are essential to improve access to information and promote positive examples to combat the COVID-19 pandemic. However, inadequate health literacy was associated with a poor understanding of COVID-19 symptoms and difficulties identifying behaviors to prevent infection. Lack of accurate information among the general public and healthcare providers is the constraint to prompt unified actions against this pandemic, highlighting the importance of effective communication on disease awareness to boost health literacy. Health care providers and health organizations should aim to assess the health literacy level of the population to be prepared and ready to deliver adequate knowledge in times of need through awareness-raising campaigns, community outreach interventions, education, and training programs as needed.

## Authors’ contributions

NH contributed to the conceptualization and study design, manuscript drafting and its editing. AMA, MKLP, AS had major roles in conceptualization and study design, helping the interpretation of the data and drafting. Rest all authors helped greatly in data extraction, interpretation, manuscript writing and revisions. All authors helped in the preparation of the final draft and manuscript revision. All authors have read and approved the submitted and revised final version of the manuscript and confirm that it is not published elsewhere and is not copied from other papers.

## Funding

There was no grant related to this study.

## Ethical approval

Not applicable. As all data used in this review have already been published, additional approval from the ethical committee was not applicable.

## Competing interests

The authors declare that they have no competing interests.

## Disclaimer

The authors claim that this manuscript has not been published in any journal and no part of this paper is copied from other sources.
